# 贝伐珠单抗在非小细胞肺癌中的应用进展

**DOI:** 10.3779/j.issn.1009-3419.2017.04.08

**Published:** 2017-04-20

**Authors:** 萍 徐, 红梅 李

**Affiliations:** 266000 青岛，青岛大学附属医院肿瘤科 Department of Oncology, Affiliated Hospital of Qingdao University, Qingdao 266000, China

**Keywords:** 贝伐珠单抗, 肺肿瘤, 不良反应, Bevacizumab, Lung neoplasms, Adverse events

## Abstract

目前非小细胞肺癌已是世界上发病率最高的癌症之一，且其发病率在逐年增加，这种现状促使人们不断寻求各种治疗手段以期延长患者的生存期，改善患者的生活质量。本综述旨在通过迄今为止有关临床数据，全面讨论贝伐珠单抗在治疗非小细胞肺癌中的应用进展，探讨该药物在临床使用中可能出现的问题、患者的更有效选择以及未来的发展方向。

## Abstract (en)

At present, non-small cell lung cancer is one of the highest incidence of cancers in the world, and its incidence increased year by year, this situation prompted people to continue to seek a variety of treatment in order to extend the survival of patients and improve the quality of life of patients. This review aims to discuss the progress of bevacizumab in the treatment of non-small cell lung cancer, through the clinical data so far to explore the clinical use of the drug, the choice of patients and the future direction of development.

现如今，肺癌特别是非小细胞肺癌（non-small cell lung cancer, NSCLC）已是世界上发病率最高的癌症之一。发病率逐年增加促使各种新的治疗方法出现，以延长患者生存期、改善患者生活质量。本文旨在讨论贝伐珠单抗在治疗晚期NSCLC中的进展，通过已有相关临床数据探讨该药物在临床实践中的应用及未来的发展方向。

## 1 贝伐珠单抗的作用机制

早在1971年，Folkman等^[1]^就已发现在肿瘤的生长、发展和转移环节中，肿瘤新生血管起到至关重要的作用。因此异常新生血管的抑制成为癌症治疗新焦点，其生成是肿瘤与环境之间相互作用的结果，有多种影响因素，其中血管内皮生长因子（vascular endothelial growth factor, VEGF）是主要相关介质。VEGF通过与VEGF受体（VEGF receptor, VEGFR）结合，促进异常肿瘤血管内皮细胞的增生、迁移并增加异常血管的通透性，而抗血管生成剂起到修剪和正常化肿瘤新生血管的作用^[2]^。贝伐珠单抗是人源化的抗VEGF药物，通过结合VEGF使VEGFR丧失活化的机会，进而发挥抗血管生成的作用^[3]^。

## 2 贝伐珠单抗在晚期NSCLC临床治疗中的应用

基于铂类化疗疗效在晚期NSCLC中似乎达到平台期^[4]^。传统化疗药本身具有抗血管生成作用，且可通过抗VEGF药物增强^[5]^。贝伐珠单抗与标准方案的联合治疗被证实可改善患者生存状况。

### 2.1 一线治疗

最早的相关研究是Margolin等^[6]^进行的Ⅰb期临床试验，试验表明贝伐珠单抗联合化疗治疗晚期癌症，患者耐受良好且没有明显的协同毒性。随后Ⅱ期临床研究将未接受过化疗的晚期NSCLC患者随机分为三组：贝伐珠单抗7.5 mg/kg或15 mg/kg联合卡铂/紫杉醇（bevacizumab+cisplatin+paclitaxel, PCB）组或单独的卡铂/紫杉醇（cisplatin+paclitaxel, PC）组。三组客观缓解率（objective response rate, ORR）、无进展生存期（progression-free survival, PFS）和总生存期（overall survival, OS）显示（表 1），联合用药组的疗效显著优于单独用药组，高剂量组的疗效优于低剂量组。最突出不良事件为出血，表现为两种临床模式：皮肤粘膜轻微出血与大咯血。严重的出血事件在鳞状细胞癌患者中更为常见。因此建议将组织学为非鳞状NSCLC（nonsquamous NSCLC, ns-NSCLC）患者作为安全风险的可接受亚群^[7]^。

**1:** 贝伐珠单抗一线治疗晚期NSCLC患者的主要临床试验 Main clinical trials of bevacizumab in chemotherapy-naive patients with advanced NSCLC

References	Treatment	Bevacizumab(mg/kg)	RR(%)	PFS(mo)	TTP(mo)	OS(mo)	Case(*n*)
Johnson *et al* 2004^[7]^	CBDCA+PTX±BEV	0	18.8		4.2	14.9	32
		7.5	28.1		4.3	11.6	32
		15.0	31.5		7.4	17.7	35
Sandler *et al* 2006^[8]^	CBDCA+PTX±BEV	0	15.0	4.5		10.3	444
		15.0	35.0	6.2		12.3	434
Yokoi *et al* 2014^[10]^	CBDCA+PEM+BEV	15.0	69.6	8.6		18.6	2, 302
Takiguchi *et al* 2015^[11]^	CBDCA+TXT+BEV	15.0	74.4	6.2		22.4	39
Seto *et al* 2014^[14]^	TARCEVA±BEV	0		9.7			77
		15.0		16.0			75
Gridelli *et al* 2016^[15]^	TARCEVA±BEV	0		10.0			100
		15.0		16.7			100
RR: response rate; PFS: progression-free survival; TTP: time to tumor progression; OS: overall survival; BEV: bevacizumab; CBDCA: carboplatin; CDDP: cisplatin; PTX: paclitaxel; TXT: docetaxel; PEM: pemetrexed; TARCEVA: erlotinib; NSCLC: non-small cell lung cancer.							

这些初步结果促使美国东部肿瘤协作组（Eastern Cooperative Oncology Group, ECOG）进行Ⅲ期临床研究。随机分配878例患者到联合治疗组与单独化疗组，结果显示联合治疗组OS显著延长，PFS明显增加，RR更高（表 1）。试验表明，PCB方案在NSCLC患者的治疗中具有显著的生存获益，同时观察到联合组比对照组发生更多的大出血事件（4.4% *vs* 0.7%），15例患者发生治疗相关性死亡，5例源于肺出血，这提示联合治疗具有增加治疗相关性死亡的风险^[8]^。

为进一步评价贝伐珠单抗联合一线标准化疗方案在临床实践中的安全性，大型Ⅳ期临床试验SAiL（*n*=2, 212）招募未治疗的局部晚期、转移性或复发性的ns-NSCLC患者，接受贝伐珠单抗加标准化疗达6个周期并单药贝伐珠单抗维持治疗直至疾病进展，结果显示严重不良事件（3级及以上）发生率较低：出血80例（4%）、肺出血15例（1%）、高血压125例（6%）、蛋白尿67例（3%）、静脉血栓栓塞症172例（8%）。57例（3%）患者死亡，血栓栓塞和出血为主要原因。该试验证实了贝伐珠单抗与各种标准化疗方案联合用于一线治疗晚期ns-NSCLC的可控性与安全性^[9]^。其他研究同样评价了贝伐珠单抗与其他标准化疗一线治疗晚期NSCLC患者的疗效性与安全性（表 1）^[10, 11]^。

BEYOND试验以中国人群为研究对象，共276例晚期ns-NSCLC患者，随机分为卡铂/紫杉醇加贝伐珠单抗（15 mg/kg）组和卡铂/紫杉醇加安慰剂组，结果显示与安慰剂组相比，中位PFS（9.2个月*vs* 6.5个月）与中位OS（24.3个月*vs* 17.7个月）均显著延长，ORR也得到明显改善（54% *vs* 26%）^[12]^。该试验进一步证实了基于贝伐珠单抗的一线治疗在中国人群中的疗效。

表皮生长因子受体（epidermal growth factor receptor, EGFR）和VEGF共享一个共同的下游通路，这表明VEGF在EGFR的封闭中起到重要作用^[13]^。基于此种情况，Seto等^[14]^与Gridelli等^[15]^分别进行了随机、开放的Ⅱ期与Ⅲ期（BEVERLY）临床研究，比较厄洛替尼联合贝伐珠单抗与单用厄洛替尼一线治疗EGFR突变的晚期ns-NSCLC患者。两项研究的结果都显示厄洛替尼联合贝伐珠单抗在PFS上有明显改善（表 1）。

### 2.2 二线治疗

一项多中心Ⅱ期试验^[16]^选取36例既往接受过至少一种化疗方案治疗的晚期NSCLC患者（包括9例脑转移患者），给予培美曲塞/奥沙利铂联合贝伐珠单抗（15 mg/kg）的治疗。中位PFS为5.8个月，中位OS为12.5个月。最常见3级毒副作用是高血压，9例（25%）脑转移患者治疗中没有脑出血发作，提示贝伐珠单抗联合化疗二线治疗晚期NSCLC耐受良好。Adjei等^[17]^评价了48例贝伐珠单抗联合培美曲塞二线治疗的晚期NSCLC患者。所有患者的疾病控制率为50%，中位OS为8.6个月，中位PFS得到改善，为4.1个月。研究还发现这个方案对于患有特定基因型SLC19A1，GGH和FPGS的晚期NSCLC患者可能特别有效。以上研究证明贝伐珠单抗和培美曲塞的组合在既往接受过治疗的晚期NSCLC患者中是有效且可耐受的。其他方案联合贝伐珠单抗的二线治疗如多西他赛/贝伐珠单抗或S-1/贝伐珠单抗^[18, 19]^，则认为尽管毒性均可耐受，但没有为患者的PFS提供任何额外的益处，两者都有待进一步调查。

### 2.3 维持治疗

维持治疗与NSCLC患者的生存改善有关。单药维持治疗已被广泛接受用于晚期ns-NSCLC，而对于贝伐珠单抗联合细胞毒性药物方案维持治疗的临床益处尚不清楚。随机研究AVAPERL评估了贝伐珠单抗/培美曲塞维持治疗与单独使用贝伐珠单抗的疗效，该研究显示，对于未进展的晚期NSCLC患者，双药维持与单独使用贝伐珠单抗相比具有显著的PFS益处（10.2个月*vs* 6.6个月；危险比0.57），总体生存率有优势^[20]^。Karayama等^[21]^将晚期ns-NSCLC患者随机分为培美曲塞/贝伐珠单抗或培美曲塞维持治疗，结果显示联合维持组的平均1年PFS率为43.9%，培美曲塞维持组为35.2%，差异不明显（*P*=0.433）。可见培美曲塞和贝伐珠单抗在晚期ns-NSCLC患者的维持治疗有效可行，但能否提高生存率有待进一步验证。

### 2.4 治疗恶性胸腔积液

恶性胸腔积液（malignant pleural effusion, MPE）是NSCLC的常见并发症，预后差。VEGF在MPE的发病机制中起关键作用，贝伐珠单抗已被证明能有效抑制胸腔积液的累积。

Du等^[22]^将72例具有MPE的NSCLC受试者随机分配到贝伐珠单抗（300 mg）加顺铂（30 mg）治疗组和单独接受顺铂（30 mg）治疗组，在治疗前后分别收集胸腔积液测其肿瘤标志物，结果显示贝伐珠单抗组的疗效显著高于顺铂组（83.33% *vs* 50.00%, *P* < 0.05），且联合治疗明显降低了胸腔积液中VEGF的水平（*P* < 0.01）。此外，联合治疗在高水平VEGF表达的患者中显示出更大的疗效（*P* < 0.01）。两组间3级/4级不良事件无明显差异。此后，两项临床试验^[23, 24]^分别对贝伐珠单抗治疗ns-NSCLC患者的MPE进行评估，均提示贝伐珠单抗对MPE患者有效且毒性可接受。

### 2.5 治疗脑转移

脑转移是NSCLC患者死亡的重要原因。最近，越来越多的研究强调贝伐珠单抗用于治疗NSCLC脑转移的疗效性与安全性。

最初试验中，脑出血的风险迫使临床医生排除了患有脑转移的NSCLC患者接受贝伐珠单抗为基础的治疗策略。Ⅱ期试验PASSPORT^[25]^与Besse等^[26]^进行的回顾性分析均包括了脑转移或用抗凝血剂治疗的患者，两项试验中观察到低的不良事件发生率，且发现脑转移患者具有独立于贝伐珠单抗的类似发生脑出血的风险，这提示贝伐珠单抗不会增加出血风险，经治疗的脑转移患者或服用抗凝血剂患者可能有使用贝伐珠单抗的资格。

前瞻性平行的Ⅱ期BRAIN试验，评估了PCB一线与厄洛替尼/贝伐珠单抗二线治疗伴有未治脑转移的Ⅳ期NSCLC患者^[27]^，这项研究中91例患者只有1例患者出现非致死性颅内出血，且联合贝伐珠单抗的治疗效果显著，特别是一线PCB组（中位PFS为6.7个月；中位OS为16.0个月）。Tang等^[28]^通过回顾性分析776例不同辅助治疗具有脑转移的晚期NSCLC患者的疗效，得出结论：化疗联合贝伐珠单抗对于脑转移的NSCLC患者更有效，对于EGFR野生型患者，化疗与贝伐珠单抗联合应用改善了PFS和OS。迄今为止已经证实了贝伐珠单抗在具有脑转移的NSCLC患者中是安全和有效的。

### 2.6 其他

Bertino等^[30]^报告了术前PCB方案用于可切除的NSCLC^[29]^，结果显示在4例入选患者中未观察到术后并发症。日本最近的一项研究同样验证了对可切除的NSLCL患者进行贝伐珠单抗联合化疗的诱导治疗是安全有效的。另有体外研究^[31, 32]^显示贝伐珠单抗联合放疗还可以增加肺癌细胞的放射敏感性，导致VEGF表达的下调，从而抑制内皮细胞中DNA双链断裂的修复，起到杀伤癌细胞作用。

## 3 不良事件及处理

贝伐珠单抗作为抗VEGF药物，除对肿瘤血管系统作用，同样会对多种组织和器官产生肿瘤外靶标效应，从而引起广泛的副反用。

### 3.1 高血压

高血压是应用贝伐珠单抗最常见的不良反应^[33]^。在NSCLC患者中，贝伐珠单抗与高血压发生风险的增加显著相关^[34]^。Dahlberg等^[35]^对ECOG 4599试验数据进行回顾性分析，发现OS和PFS与治疗期间高血压的发生率之间存在正相关。而另一项Ⅲ期临床试验^[36]^则认为早期治疗相关的血压升高并不能预测贝伐珠单抗基于PFS或OS结果的临床益处。因此高血压的临床意义是不确定的，有待进一步研究。而早期监测和有效管理高血压是安全使用贝伐珠单抗的重要步骤。

### 3.2 肾功能损害

赵静等^[37]^通过分析4例贝伐珠单抗诱导的肾功能损害患者的临床资料和病理资料，发现在贝伐珠单抗诱导的肾功能损伤中，最常见的临床表现是蛋白尿（>3.5 g/d），其他症状包括微血尿，恶性高血压，血清肌酐水平升高伴随急性肾衰竭和无尿。贝伐珠单抗停药后的预后相对较好，因此在治疗期间应密切监测尿蛋白排泄和肾功能，以早期识别任何肾损伤。

### 3.3 出血

临床中可观察到的常见出血事件有皮肤黏膜出血、鼻出血、消化道出血、肺出血及脑出血。一项荟萃分析^[38]^显示贝伐珠单抗参与的化疗显著增加了高度出血的风险，且风险可能是剂量依赖性的。严重肺出血是贝伐珠单抗治疗晚期NSCLC相关的罕见但严重潜在不良事件，应对所有接受贝伐珠单抗的NSCLC患者进行个体化的风险-益处评估^[39]^，在使用贝伐珠单抗期间的抗凝治疗也需要进行严密的凝血功能监测。

### 3.4 其他不良事件报道

有研究^[40]^显示贝伐珠单抗联合化疗的治疗与动、静脉血栓栓塞风险增加相关。贝伐珠单抗还可以单独诱导颌骨坏死，并且与双磷酸盐的组合可以增加其风险^[41]^。有证据证明贝伐珠单抗还可引起输注反应^[42]^、溃疡性结肠炎^[43]^、心脏毒性及慢性间质性肺炎^[44]^。与常规治疗相比，贝伐珠单抗增加了癌症的伤口愈合并发症的发生率，特别是对于结肠癌患者^[45]^，但对NSCLC的不利影响尚无定论。

## 4 待解决的问题

### 4.1 药物剂量与应用方式

Heist等^[46]^发现贝伐珠单抗治疗后血管通透性的过度降低，可能对NSCLC患者的联合治疗效果产生负面影响。另一项研究^[47]^显示内分泌器官的血管系统对抗VEGF治疗比肿瘤血管系统更加敏感，低剂量的VEGF阻断不但导致内分泌器官的功能损害，还对肿瘤血管系统无效。这两项研究都表明需要对贝伐珠单抗在肺癌治疗中的最适剂量进行进一步研究与验证。一项最新试验^[48]^表明在癌症中停止抗VEGF的治疗会促进癌症的转移，这一发现对抗血管生成疗法提出了不间断和持续应用的新挑战。另一项试验中，Xiao等^[49]^通过在人A549肿瘤模型中使用Re-188（一种β放射性核素）标记的贝伐珠单抗进行放射免疫治疗，发现Re-188增强了贝伐珠单抗在非小细胞肺癌中的抑制作用，这提示了在NSCLC治疗中贝伐珠单抗应用的潜在治疗策略。

### 4.2 有效的监测标准

NSCLC患者的治疗疗效通过实体瘤疗效评价标准（Response Evaluation Criteria in Solid Tumors, RECISTs）标准进行评价，但临床观察到使用贝伐珠单抗治疗的NSCLC患者，部分可发生肿瘤空洞且大多数空洞随后被填充，影像上可显示为3种放射图像^[50]^。因此，鉴于每个患者的治疗方法的多样性，对于有贝伐珠单抗参与治疗NSCLC患者，应寻找个体化的治疗疗效标准。

## 5 展望

贝伐珠单抗打破了常规化疗在晚期NSCLC治疗中可以实现的OS极限，特别是证据最有力的PCB方案。抗血管生成的治疗方式将人们的视角从消灭肿瘤本身转移到控制肿瘤微环境，这也符合当下“带瘤生存”的观点。即便仍然存在很多疑问，例如贝伐珠单抗的耐药及作用机制不完全明了等，但不可否认的是，贝伐珠单抗确实为一些患者带来生存获益，只是需要更多的试验来提供更有效使用该药物的详细信息，如更能获益人群的选择和疗效上的改善等。
